# 

**Published:** 1914-06-13

**Authors:** 


### CONTENTS.

PAGE

**The Laymen's Influence in Public Medicine** 281

**The Hampshire Tuberculosis Officership** ... 282

**Hospital and Institutional News...** ... ... 283

The New Superintendent at North Evington Infirmary—The Qualities Needed in an Infirmary Superintendent—New Treasurers of St. George's Hospital—The Latest Method in Hospital Histories—The Homœopathic Hospital's History—The Secret of Hospital Advertising—The Hospital Standard in Advertising—The Blackpool Hospital Deadlock — Legacy of £30,000 for a Practitioner—The Hospital Sunday Crisis—German National Insurance Institutional Statistics—The Hospital Leaven in Prison Cells—Effects of the Hospital Standard—Long Hours and Short Holidays—Lebanon Hospital for Mental Cases—Competitive Designs at Burnley—Queen Alexandra and Ancoats Hospital—A New Children's Hospital for Dundee—The Mission Hospital Problem—Devizes Town Hall as a Hospital—Sir William Osler's Crusade—Next Week's Conference at Newcastle—Seasonal Lunacy and Overcrowded Wards—The Dispensers' Half-holiday—The Deficiency in the Drug Fund—This Week's Drug Market.

PAGE

**Mental Symptoms and Patients' Thoughts** ... 289

The Methods of Psycho-analysis.

**Trade Hostility to Hospital Research** ... ... 290

The Ideal Antiseptic for Obstetric Work.

**The School Doctor and Skin Disease** ... ... 291

Symptoms that may Lead to the Police Court.

### CONTENTS—(*continued*).

PAGE

**Institutional Residents and Recreation** ... 292

The Escape from the Common-room Atmosphere.

**Colloid Metals in the Clinical Laboratory** ... 292

**Reports on Hospitals of the United Kingdom**— 293

By SIR HENRY BURDETT, K.C.B., K.C.V.O.

Kent County Ophthalmic Hospital, Maidstone.

**The Prevention of Typhoid Fever** ... ... 293

**Radium for the Voluntary Hospitals** ... ... 294

A Radium Week by Artizan Workers.

**New Developments at "The London"** ... 295

Mr. W. T. Paulin on the Effect of the Insurance Act.

**The London Panel Committee** ... ... ... 295

Co-operation Centres, Prescribing and Fees.

**Economy in the Generation and Use of Steam** ... ... ... ... ... ... ... 296

II.—The Principal Apparatus Described.

**Round the World** ... ... ... ... ... ... 297

Fellow-Workers and their Doings.

PAGE

**Should Surgeons Mask While Operating?** ... 298
The Deadliness of a Sneeze.
**Pharmacists and the Cocaine Snuff Habit** ... 298
**The Department of Modern Nursing** ... ... 299
III.—The London Hospital.
**Medical Appointments** ... ... ... ... ... 301
**The Editor's Letter-Box** ... ... ... ... 303
Institutional Defalcations: The Assistant-Secretary's Responsibility—A Question of Professional Conduct—The Risk of Premature Burial—The Retrograde Policy of the Bath Guardians.
**The "Jenner" Lifting Pole** ... ... ... 304
**The Patients' Column** ... ... ... ... ... 304
**Legal Notes for Institutional Workers** ... 305
**The Profession of Medicine** ... ... ... 306
Major Surgery and Human Character: A Novel.
Old-age and Eyesight.
The Romance of Camphor.
**The National Medical Union (Official)** ... ... 308
**The Institutional Worker** ... ... (Suppl.) 1
Hospital Supplies: Purchase by Contract or in Open Market.

